# Purinergic signaling in osteoarthritis: Mechanistic insights into pathogenesis and therapeutic targeting

**DOI:** 10.1016/j.gendis.2025.102002

**Published:** 2025-12-23

**Authors:** Hongliang Li, Tianqi Wang, Zi Wang, Jincen Hou, Zhong Li, Jiyuan Yan

**Affiliations:** a Department of Orthopaedics, The Affiliated Hospital of Southwest Medical University, Luzhou, Sichuan 646000, China; b Sichuan Provincial Laboratory of Orthopaedic Engineering, The Affiliated Hospital of Southwest Medical University, Luzhou, Sichuan 646000, China; c Stem Cell Immunity and Regeneration Key Laboratory of Luzhou, The Affiliated Hospital of Southwest Medical University, Luzhou, Sichuan 646000, China; d Department of Orthopaedic Surgery, Yong Loo Lin School of Medicine, National University of Singapore, 119228, Singapore

**Keywords:** Adenosine, Extracellular nucleotide, Osteoarthritis, P1 receptors, P2 receptors, Purinergic signaling

## Abstract

Osteoarthritis (OA) is a degenerative joint disease driven by a complex interplay of inflammation, extracellular matrix degradation, subchondral bone remodeling, and chronic pain. Purinergic signaling has emerged as a key regulator of OA pathogenesis, where dysregulated extracellular nucleotide-mediated P2 receptor activation and impaired adenosine-mediated P1 receptor signaling disrupt joint homeostasis. Excessive activation of P2X and P2Y receptors amplifies inflammatory cascades, promotes chondrocyte apoptosis, enhances matrix metalloproteinase activity, and sensitizes nociceptive pathways, while reduced P1 receptor signaling, particularly via A2A and A3, compromises anti-inflammatory and chondroprotective mechanisms. Additionally, disruptions in extracellular nucleotide metabolism exacerbate disease progression by perpetuating synovial fibrosis, cartilage destruction, and persistent pain. This review provides a mechanistic overview of purinergic receptor dysregulation in OA, detailing its roles in synovial inflammation, cartilage homeostasis, subchondral bone remodeling, and pain transmission. Furthermore, this review explores emerging therapeutic strategies targeting purinergic receptors, particularly P2 receptor antagonists and P1 receptor agonists, which are being developed as selective ligands to restore joint homeostasis and attenuate disease progression. Understanding the intricate molecular crosstalk between the P2 and P1 receptor pathways will be critical for the development of precision therapies aimed at modifying OA pathophysiology and improving clinical outcomes.

## Introduction

Osteoarthritis (OA) is one of the most prevalent degenerative joint disorders and a leading cause of disability.^1^, ^2^, ^3^ It is characterized by progressive articular cartilage degradation, subchondral bone sclerosis, synovial inflammation, and osteophyte formation, ultimately leading to joint pain, stiffness, and functional impairment.^4^^,^^5^ Although OA is traditionally regarded as a mechanical wear-and-tear condition, accumulating evidence suggests that OA is a multifactorial disease driven by a complex interplay of biomechanical stress, low-grade chronic inflammation, metabolic dysregulation, and oxidative stress.^6^^,^^7^ Epidemiological data indicate that OA disproportionately affects women, likely due to hormonal influences, and is strongly associated with aging, obesity, and prior joint trauma, all of which contribute to alterations in joint homeostasis and exacerbate inflammatory and catabolic processes.^8^^,^^9^

Recent advances have identified the purinergic signaling system as a critical regulatory network in OA pathogenesis. This evolutionarily conserved extracellular nucleotide-based signaling system plays a central role in modulating chondrocyte function, extracellular matrix homeostasis, inflammation, mechanotransduction, and clinical symptoms.^10^^,^^11^ Purinergic receptors, widely expressed in musculoskeletal tissues, are classified into two major families: P1 receptors, which are G protein-coupled adenosine receptors (A1, A2A, A2B, and A3), and P2 receptors, which include ionotropic P2X receptors (P2X1–P2X7) and metabotropic P2Y receptors (P2Y1, P2Y2, P2Y4, P2Y6, P2Y11, P2Y12, P2Y13, and P2Y14).^12^^,^^13^ These receptors respond to extracellular nucleotides such as adenosine triphosphate (ATP) and adenosine, coordinating a broad spectrum of physiological and pathological processes in joint tissues.^14^

Among the diverse purinergic receptor subtypes, A2A receptor activation has been shown to exert chondroprotective effects by mitigating oxidative stress, enhancing autophagy, and preserving cartilage integrity, highlighting its potential as a therapeutic target.^15^, ^16^, ^17^ In contrast, P2X7 receptor activation has been implicated in OA pathogenesis due to its role in promoting pro-inflammatory cytokine release and accelerating cartilage degradation.^18^^,^^19^ P2Y receptors further contribute to disease progression by regulating chondrocyte proliferation, matrix turnover, and inflammatory mediator production, underscoring the multifaceted and interconnected roles of purinergic signaling in joint degeneration.^20^

This review provides a comprehensive synthesis of the current knowledge on purinergic receptor modulation in OA, emphasizing its role in disease progression and therapeutic potential. By integrating recent findings and identifying key research priorities, this article aims to enhance the understanding of purinergic signaling in OA pathophysiology and explore its translational potential as a therapeutic target. The following sections examine the principal risk factors of OA, highlight the critical role of the purinergic receptor system in joint pathology, and discuss emerging advances in purinergic signaling research and therapeutic interventions.

## Osteoarthritis as a multifactorial disease and its modulation by purinergic receptors

### Multifactorial determinants of OA pathogenesis

Osteoarthritis (OA) is traditionally seen as a passive consequence of mechanical wear.^6^ However, accumulating evidence redefines it as an active, multifactorial disease driven by interrelated inflammatory, metabolic, and biomechanical processes that disrupt joint homeostasis.^4^^,^^6^^,^^21^ OA involves not only cartilage erosion but also coordinated dysfunction across multiple joint compartments, including synovium, subchondral bone, and periarticular structures.^4^^,^^22^ This paradigm shift reframes OA from symptom management to targeting underlying pathogenic mechanisms.

**Figure 1:**
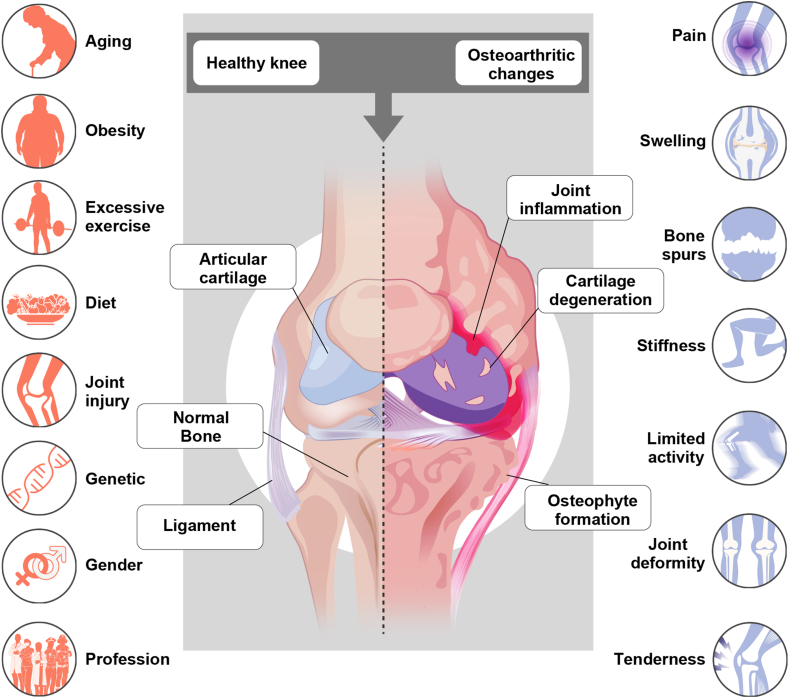
Pathophysiology of osteoarthritis—risk factors, structural degeneration, and clinical implications. This figure delineates the complex pathophysiology of osteoarthritis (OA), highlighting the interrelationship between risk factors, structural joint changes, and clinical manifestations. Key etiological factors—including aging, obesity, joint injury, genetic predisposition, and aberrant mechanical loading—drive the initiation and progression of OA by disrupting joint homeostasis. In a physiologically healthy joint, articular cartilage, synovial fluid, ligaments, and subchondral bone function synergistically to facilitate smooth articulation and load-bearing capacity. In OA, progressive cartilage matrix degradation, osteophyte formation, subchondral bone sclerosis, and synovial inflammation result in irreversible structural damage, leading to chronic pain, joint stiffness, swelling, and functional impairment. The interplay between biomechanical stressors, inflammatory cascades, and metabolic dysregulation underscores the multifactorial nature of OA, emphasizing its progressive and debilitating course.

The rising OA burden, particularly in aging and obese populations, demands a shift in research and treatment priorities.^23^, ^24^, ^25^ Nearly one-third of adults over 65 are affected, with higher prevalence in women due to hormonal modulation of joint physiology.^8^^,^^26^ Age and obesity contribute to mitochondrial dysfunction, cellular senescence, and immunosenescence, fostering a pro-inflammatory milieu that accelerates disease progression (Fig. 1).^27^, ^28^, ^29^ Obesity is a major driver of OA, influencing disease progression through both biomechanical and metabolic mechanisms.^30^^,^^31^ While excessive joint loading has historically been considered the primary link between obesity and OA, recent evidence highlights the pivotal role of metabolism-related inflammation in disease pathology.^32^^,^^33^ Obesity is associated with the systemic release of pro-inflammatory adipokines, which are related to OA pain and modulate chondrocyte metabolism, synovial inflammation, and extracellular matrix (ECM) degradation.^30^^,^^34^ In addition, obesity is frequently accompanied by insulin resistance, dyslipidemia, and chronic low-grade inflammation, collectively termed metabolic syndrome (MetS), all of which may exacerbate OA pathogenesis by increasing oxidative stress and impairing cartilage repair mechanisms.^35^^,^^36^

Beyond aging and metabolic dysfunction, additional risk factors, such as joint trauma, repetitive mechanical stress, and genetic predisposition, contribute to OA as an active disease process rather than a linear degenerative event (Fig. 1).^37^, ^38^, ^39^ Cartilage damage often occurs in post-traumatic OA, a condition commonly observed in people with meniscal damage.^38^^,^^40^ Similarly, joint malalignment and abnormal loading patterns promote disease onset by inducing chronic biomechanical stress on articular cartilage, triggering an active remodeling response that paradoxically accelerates joint destruction.^41^ Genetic susceptibility also plays a role, with polymorphisms in genes of cartilage protection and gene modification, which may contribute to altered joint metabolism and increased disease susceptibility.^37^

### Characterization of purinergic receptor families and signaling properties

Purinergic signaling regulates essential physiological functions such as neurotransmission, immune modulation, vascular homeostasis, and tissue remodeling.^42^, ^43^, ^44^ It operates via extracellular nucleotides and nucleosides—including ATP, adenosine diphosphate (ADP), uridine triphosphate (UTP), uridine diphosphate (UDP), and adenosine—which activate specific purinergic receptors.^45^^,^^46^ These receptors are categorized into P1 receptors, activated by adenosine and primarily involved in anti-inflammatory responses, and P2 receptors, responsive to a broad spectrum of extracellular nucleotides.^47^^,^^48^ P2 receptors include ionotropic P2X receptors, which function as ligand-gated ion channels mediating calcium influx and inflammation, and metabotropic P2Y receptors, which are GPCRs that regulate metabolism, immune activity, and tissue remodeling.^49^^,^^50^

Extracellular nucleotide availability is tightly regulated by ectonucleotidases, primarily CD39 and CD73, which catalyze the stepwise degradation of ATP into ADP, AMP, and ultimately adenosine.^51^^,^^52^ This enzymatic cascade finely modulates purinergic signaling by controlling receptor activation dynamics. Adenosine exerts diverse effects via P1 receptors, depending on the subtype and tissue context.^53^^,^^54^ A1 and A3 receptors, coupled to Gi proteins, inhibit adenylyl cyclase, reduce cAMP levels, and modulate pain and inflammation (Table 1, Table 2).^55^^,^^56^ In contrast, Gs-coupled A2A and A2B receptors elevate cAMP, mediating anti-inflammatory, vasodilatory, and cytoprotective responses.^57^^,^^58^

**Table 1:** Some specific roles of different purinergic receptor types.

Type	System	Role in different system	Reference
A1R	Nervous system	Agonists exhibit significant cerebroprotective efficacy in acute ischemic stroke, highlighting its potential therapeutic applications.	98
		Activation of A1R decreases seizure activity in animal models.	111
		A1R signaling activation contribute to spinal cord stimulation-induced inhibition of spinal nociceptive transmission.	56
A2AR	Nervous system	Adenosine emerges as a promising candidate molecule for activating indirect pathway neurons of the nucleus accumbens (NAc) that express A2AR, thereby inducing slow-wave sleep.	197
		Early increase in A2AR expression amid persistent amyloid pathology worsens memory deficits.	101
A2BR	Hepatic and renal system	A2BR promotes penile rehabilitation in refractory erectile dysfunction by alleviating hypoxia and fibrosis, underscoring its potential therapeutic role in musculoskeletal tissue repair.	118
P2X1	Hepatic and renal system	In renal ischemia/reperfusion injury, P2X1 activation enhances ATP release, thereby promoting the formation of neutrophil traps	119
	Gastrointestinal system	P2X1 inhibitor improved the therapeutic efficacy of anti-TNF-α therapy in a colitis mouse model.	107
P2X3	Nervous system	Activated by ATP, offering potential for innovative treatments of chronic pain.	126
P2X4	Gastrointestinal system	Microbiota dysbiosis induced by the P2X4 influences the development of colitis.	108
	Hepatic and renal system	The lack of P2X4 may help reduce liver injury by limiting inflammation and oxidative stress.	120
		Treatment of the P2X4 antagonist alleviates collagen-induced arthritis in mice by suppressing the Th17 cells proliferation and activation.	94
P2X5	Musculoskeletal system	Shown to regulate osteoclast maturation.	95
P2X6	Hepatic and renal system	High expression of P2X6 in the T24 cell line may be a potential predictive marker for improved survival outcomes in bladder cancer patients.	121
P2X7	Cardiovascular system	P2X7 deficiency alleviates cardiac dysfunction, suggesting its potential as a therapeutic target for hypertensive heart failure.	104
P2Y1	Cardiovascular system	P2Y1 improves arterial dysfunction in endothelial cell autophagy.	44
P2Y2	Respiratory system	P2Y2 leads to an increase in endogenous ATP levels, which is linked to the development of lung inflammation.	103
	Musculoskeletal system	Activation of P2Y2 under mechanical loading initiates a negative feedback loop that attenuates the osteocyte response to sustained mechanical stimulation.	159
P2Y6	Nervous system	P2Y6 impacts myeloid cell immune function during epileptogenesis.	99
	Gastrointestinal system	Activation of P2Y6 protects the intestine from inflammation in colonic epithelial cells.	122
P2Y11	Other system	Autocrine signaling through the P2Y11 modulates the migration of CD4^+^ T cells.	166
		P2Y11 triggers IL-1 receptor signaling thereby blocking TNF-α secretion and aiding in the resolution of inflammation.	164
P2Y12	Cardiovascular system	P2Y12 enhancing platelets activation, aggregation, degranulation, and change.	163
P2Y13	Hepatic and renal system	P2Y13 serve as a pharmacological approach to prevent steatotic liver disease.	72
P2Y14	Musculoskeletal system	P2Y14 bind with inflammasome NLRP3 show a significant role in the pathogenesis of acute gouty arthritis.	167
	Respiratory system	Deletion of P2Y14 reduces airway immune cell infiltration and airway hyperresponsiveness to asthma.	114

**Table 2:** Purinergic receptors have direct and possibly indirect effects on OA.

Receptor Type	Tissue Distribution	Mechanism	Pathological Process	Reference
A1R	Neurons	Suppression of nociceptive transmission	Reduced pain after nerve injury	56
A2AR	Cartilage	Maintain or enhance mitochondrial function	Decrease reactive oxygen species	15
A3R	Cartilage	Inhibition of ROS/NLRP3/GSDMD signalling	Attenuate progression and relieve pain perception	90
P2X3	Neurons	Sensitization of ATP on sensory neurons in the periphery	Contribution to pain	126
P2X7	Cartilage	NF-κB/NLRP3 crosstalk	Induce pyroptotic inflammation and cartilage degradation	133
P2Y1	Synovium	G(αq)/Ca^2+^-NF-κB/NFAT pathway in macrophage	Neutrophil recruitment	154
P2Y2	Bone	Altered osteocyte sensitivity to mechanical strain	Inhibiting response to sustained mechanical loading	159

P2X receptors (P2X1–P2X7) mediate rapid ATP-induced cation influx—primarily Ca^2+^ and Na^+^—and are essential for neurotransmission, mechanosensation, immune activation, and pain perception.^59^, ^60^, ^61^ P2X7 is a key inflammatory target, as its activation induces NLRP3 inflammasome assembly and promotes IL-1β and TNF-α release.^62^^,^^63^ P2X3, predominantly expressed in sensory neurons, is critical for nociceptive signaling.^64^^,^^65^

P2Y receptors mediate slower, sustained nucleotide signaling by coupling to Gq, Gs, or Gi proteins, thereby regulating intracellular calcium flux, PLC activation, and downstream pathways.^66^^,^^67^ P2Y2, P2Y4, and P2Y6 activate PLC–IP_3_ signaling to induce Ca^2+^ release, while P2Y11 uniquely engages both the Gq and Gs pathways, coordinating metabolic and inflammatory responses.^68^, ^69^, ^70^ Gi-coupled P2Y12, P2Y13, and P2Y14 receptors suppress cAMP production and regulate platelet aggregation, immune cell trafficking, and lipid metabolism.^71^, ^72^, ^73^

### Historical progression of purinergic receptor in OA

Among the emerging regulators of OA as an active and dynamic disease is the purinergic signaling system, which orchestrates fundamental cellular processes, including inflammation, chondrocyte metabolism, and mechanotransduction.^11^^,^^74^^,^^75^ Purinergic receptors, classified into P1 (adenosine-sensitive) and P2 (extracellular nucleotide-sensitive) receptors, mediate extracellular nucleotide signaling and have been implicated in both protective and pathological responses in OA.^11^^,^^76^ The P2X7 receptor, a key modulator of inflammasome activation, promotes synovial inflammation and facilitates pro-inflammatory cytokine release, exacerbating joint tissue destruction.^19^^,^^77^ In contrast, activation of the A2A receptor has been shown to exert chondroprotective effects by enhancing autophagy, reducing oxidative stress, and preserving ECM integrity.^78^^,^^79^ Dysregulation of these receptors contributes to an imbalance in joint homeostasis, shifting the equilibrium toward a pro-inflammatory and catabolic state.

Advances in purinergic biology have marked distinct stages in our understanding of osteoarthritis pathogenesis. In the 1970s–1990s, the concept of purinergic receptors was established and extracellular ATP, via P2 receptors, was shown to promote PGE_2_ release in chondrocytes, synergizing with cytokines such as IL-1β and TNF-α.^80^, ^81^, ^82^ By 1999, human chondrocytes were confirmed to express A2A, A2B, and P2Y2 receptors, linking P2Y2 to inflammatory prostaglandin release.^83^ In the 2000s, attention shifted to adenosine receptors, with A2A elevating cAMP, suppressing NO, and protecting cartilage, while A3 and A2A up-regulation by physical stimuli enhanced chondrocyte survival.^84^^,^^85^ During the 2010s–2020s, functional specificity became clear: A2A deficiency caused spontaneous OA, A3AR agonists attenuated cartilage degeneration and pain, and P2X7 emerged as a central inflammatory switch driving NF-κB/NLRP3 pyroptosis.^79^^,^^86^, ^87^, ^88^, ^89^, ^90^, ^91^

### Receptor-mediated pathways shaping OA subtypes

Across metabolic OA, excess nutrients and metabolic syndrome drive persistent low-grade inflammation, with A2AR exerting anti-inflammatory and autophagic protection, while P2X7 activates the inflammasome and promotes matrix degradation.^75^ Within post-traumatic OA, A3R activation reduces cartilage damage, pain, and pyroptosis via the suppression of ROS/NLRP3/GSDMD signaling, and A2AR ligation prevents matrix degradation and aberrant chondrocyte differentiation.^78^^,^^90^ During obesity-induced OA, A2AR stimulation activates FoxO1/3, enhances autophagic flux, restores mitochondrial function, and decreases apoptosis, thereby sustaining cartilage homeostasis.^16^ For temporomandibular joint osteoarthritis (TMJ-OA), ADP-induced activation of P2Y1, P2Y12, and especially P2Y13 upregulates MCP-1/CCL2 in synoviocytes through MEK/ERK signaling, driving inflammatory infiltration.^92^ At the level of lumbar facet joint OA, P2Y12 limits IL-1β–induced apoptosis via PI3K/AKT activation, maintaining COL2 expression and restraining MMP13 up-regulation.^93^

### Purinergic receptor networks linking osteoarthritis to multi-organ regulation

#### Purinergic signaling in systemic pathways converging on OA

**Figure 2:**
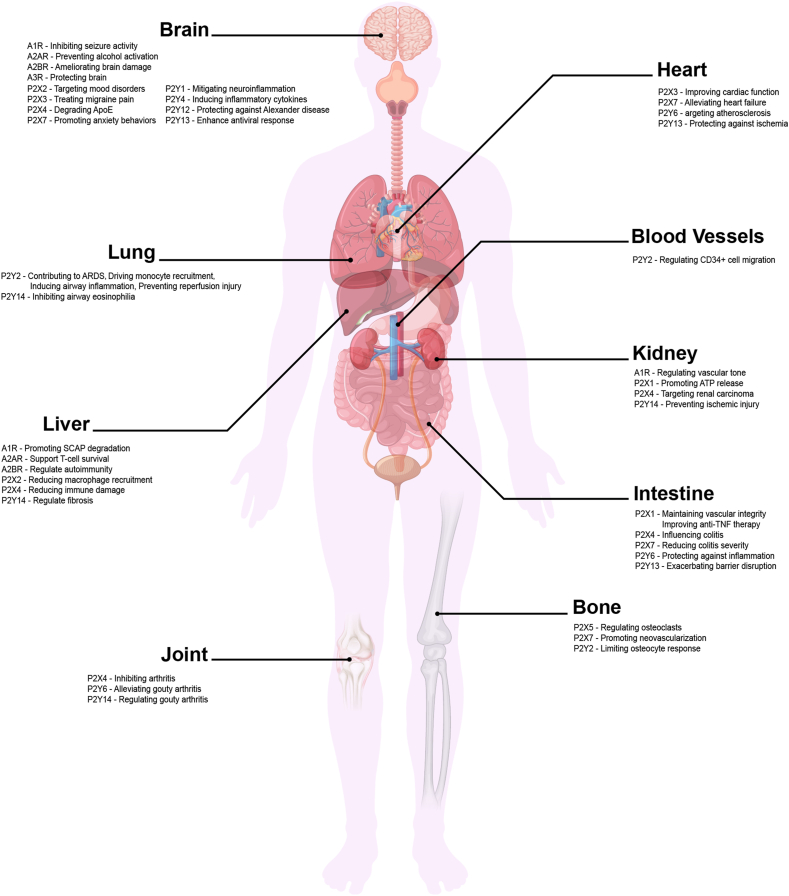
Systemic distribution and functions of adenosine (P1) and purinergic (P2X and P2Y) receptors. This figure illustrates the widespread distribution and diverse physiological roles of adenosine (P1) and purinergic (P2X and P2Y) receptors across multiple organ systems. In the nervous system, A1R suppresses epileptic activity, A2AR protects against alcohol-induced neurotoxicity, P2X2 and P2X4 influence neuropsychiatric disorders, and P2Y12 is linked to Alzheimer's disease. In the cardiovascular system, P2X3 enhances cardiac function, while P2Y6 is involved in atherosclerosis. In the respiratory system, P2Y2 promotes airway inflammation, whereas P2Y14 reduces eosinophilic responses. In the digestive system, A2AR regulates immune tolerance, P2X4 mitigates immune-mediated damage, and P2Y14 modulates fibrosis. In the urinary system, P2X1 contributes to bladder ATP release, while P2Y14 protects against renal ischemia. In the musculoskeletal system, P2X4 influences arthritis, and P2Y2 regulates osteocyte function. This figure highlights the system-specific functions of purinergic signaling in health and disease, emphasizing its potential as a therapeutic target.

In the musculoskeletal system, inhibition of P2X4 alleviates collagen-induced arthritis by suppressing Th17 cell expansion, while P2X5 regulates osteoclast maturation (Fig. 2 and Table 1).^94^^,^^95^ ATP-mediated P2X7 activation promotes inflammation, osteoclastogenesis, and extracellular matrix degradation, whereas A2AR activation confers chondroprotection, anti-inflammatory effects, and bone repair.^16^^,^^19^^,^^96^ The bone microenvironment is particularly sensitive to purinergic signaling, with P2 receptors modulating osteoblast differentiation and bone resorption.^96^^,^^97^

In the nervous system, P2X- and P2Y-mediated glial activation and neuroimmune signaling contribute to pain sensitization, a process central to OA (Table 1).^98^, ^99^, ^100^, ^101^ In the respiratory system, inhibition of P2X3 alleviates bronchoconstriction and cough, mechanisms analogous to nociceptive sensitization in OA, while P2Y2-driven ATP release fuels LPS-induced inflammation resembling synovial cascades (Fig. 2 and Table 1).^102^^,^^103^ In the cardiovascular system, P2X7 promotes cardiac remodeling and ferroptosis, paralleling subchondral bone changes and chondrocyte death in OA (Table 1).^104^ Hepatic and renal evidence further links P2X7 to hepatic fibrosis and non-alcoholic fatty liver disease (NAFLD), pathologies that share metabolic and fibrotic features with OA.^105^^,^^106^ Similarly, in the gastrointestinal tract, P2X1 and P2X4 regulate inflammation and microbial balance, processes increasingly recognized in OA's metabolic–immune axis (Fig. 2 and Table 1).^107^^,^^108^

#### Roles of purinergic receptor across representative organ systems

Beyond OA-related mechanisms, purinergic receptors perform diverse functions across organ systems. In the nervous system, A1 and A2A regulate excitability, ischemic tolerance, and memory, while dysregulation contributes to Alzheimer's, Parkinson's, epilepsy, and multiple sclerosis (Table 1).^101^^,^^109^, ^110^, ^111^ In the lungs, P1 receptors promote airway relaxation and anti-inflammatory signaling, while P2Y14 deficiency reduces eosinophilia and hyperresponsiveness in asthma (Table 1).^112^, ^113^, ^114^ In the cardiovascular system, A1 activation mediates bradycardia and cardioprotection, P2X receptors drive vasoconstriction, and P2Y receptors support vasodilation and platelet aggregation, maintaining vascular homeostasis.^66^^,^^115^, ^116^, ^117^ In the hepatic and renal systems, A2B activation aids erectile recovery, P2X1 enhances neutrophil glycolysis in renal ischemia, P2X4 deficiency protects against liver injury, and P2X6 serves as a prognostic marker in bladder cancer (Fig. 2 and Table 1).^118^, ^119^, ^120^, ^121^ In the gastrointestinal tract, P2Y receptors preserve mucosal barrier integrity and immune balance, thereby mitigating chronic intestinal inflammation (Table 1).^122^

### Purinergic receptor dysregulation in OA pathogenesis

Purinergic signaling governs inflammation, metabolism, and tissue remodeling across organ systems, with a critical balance between extracellular nucleotide-driven P2 receptor activation and adenosine-mediated P1 signaling essential for homeostasis. The following sections examine the roles of P2X, P2Y, and P1 receptors in OA pathogenesis, focusing on their contributions to synovial inflammation, chondrocyte dysfunction, matrix destruction, bone changes, and pain, thereby identifying potential therapeutic targets.

### P2X receptors in OA pathogenesis

P2X receptors are ATP-gated ion channels experimentally validated as mediators of mechanotransduction, synovial inflammation, chondrocyte apoptosis, ECM degradation, and nociceptive sensitization in OA.^18^^,^^123^ By driving Na^+^, Ca^2+^, and K^+^ flux, they are mechanistically shown to function under mechanical and inflammatory stress.^59^^,^^124^ In OA, ATP release from damaged cartilage has been observed in models, sustaining receptor activation and perpetuating inflammation and pain.^125^ Among these subtypes, direct evidence indicates that P2X7 drives inflammatory and catabolic pathways, while P2X3 mediates pain and central sensitization (Fig. 4).^19^^,^^126^

**Figure 3:**
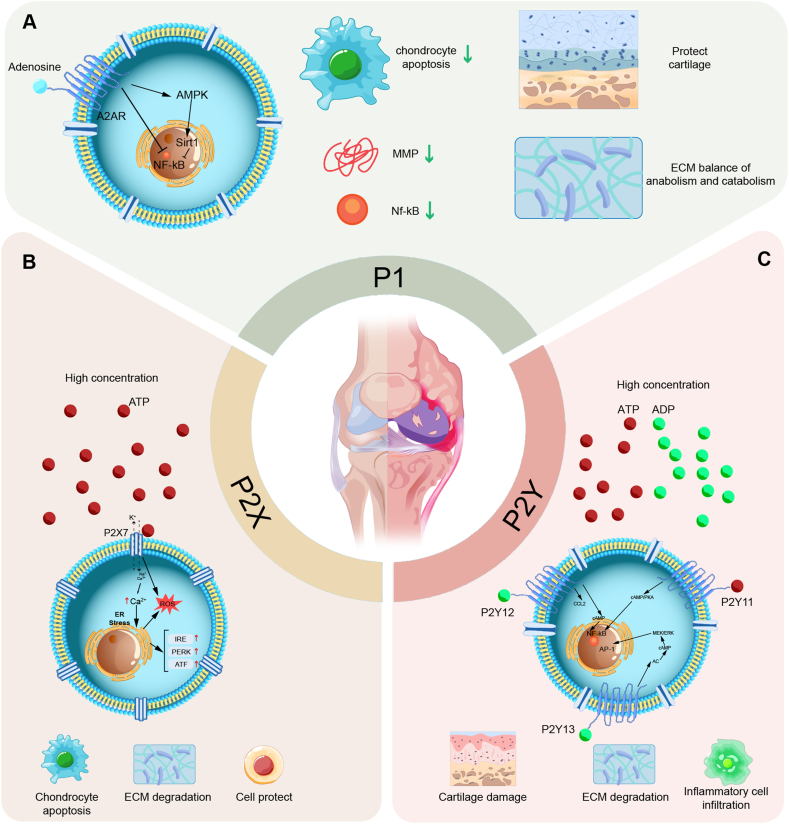
Purinergic signaling in joint homeostasis and osteoarthritis progression. This figure illustrates the regulatory roles of P1, P2X, and P2Y receptors in joint physiology and osteoarthritis (OA) pathogenesis. **(A)** A2A receptor (A2AR) activation by adenosine suppresses nuclear factor kappa-B (NF-κB) signalling, reduces matrix metalloproteinase (MMP) expression, and prevents chondrocyte apoptosis, thereby preserving cartilage integrity and extracellular matrix homeostasis. **(B)** P2X7 receptor activation by ATP induces endoplasmic reticulum stress, increasing PERK and activating transcription factor (ATF) expression, which may contribute to OA progression. **(C)** ATP/ADP serve as activating ligands for P2Y receptors, with P2Y11, P2Y12, and P2Y13 modulating OA progression through NF-κB interactions. These signaling pathways highlight the complex and dynamic role of purinergic signaling in joint health and disease.

**Figure 4:**
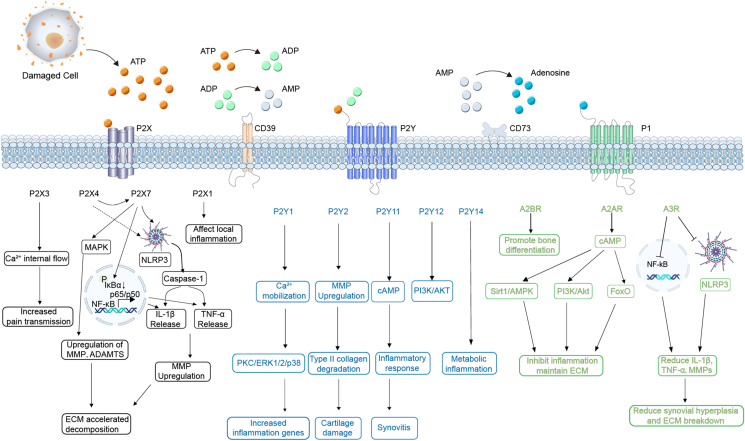
Purinergic receptor signaling pathways in OA. This schematic illustrates the activation and downstream signaling of P2X, P2Y, and P1 purinergic receptors in the joint microenvironment. ATP released from damaged cells activates P2X receptors (P2X1, P2X3, P2X4, P2X7), leading to calcium influx, NF-κB and MAPK activation, inflammasome assembly, and subsequent release of IL-1β, TNF-α, and MMPs, thereby promoting extracellular matrix (ECM) degradation, synovial inflammation, and pain transmission. P2Y receptors (P2Y1, P2Y2, P2Y11, P2Y12, P2Y14) regulate diverse processes, including calcium mobilization, MMP up-regulation, PI3K/AKT and ERK1/2/p38 activation, collagen degradation, inflammatory cytokine production, and metabolic inflammation. Adenosine generated via CD73 activates P1 receptors (A2AR, A2BR, A3R), which exert protective functions by enhancing the cAMP, Sirt1/AMPK, and FoxO pathways, inhibiting NF-κB signaling, promoting bone differentiation, reducing inflammatory cytokines, and preserving ECM integrity. Together, the figure highlights the balance between deleterious ATP–P2 receptor signaling and protective adenosine–P1 receptor signaling in osteoarthritis pathogenesis.

#### P2X7 receptor: a central mediator of synovial inflammation and cartilage breakdown

P2X7 has been confirmed in cell and animal studies to be central to OA pathogenesis.^19^ At high ATP levels, it experimentally forms pores, inducing pyroptosis, apoptosis, or necrosis.^127^^,^^128^ Chronic ATP functions as a danger-associated molecular pattern (DAMP), activating P2X7, which has been shown to trigger NLRP3 inflammasome assembly, caspase-1 activation, and IL-1β/TNF-α release, thereby accelerating cartilage breakdown in OA models.

##### Role in synovial inflammation and NLRP3 inflammasome activation

P2X7 is experimentally established as a key regulator of NLRP3 inflammasome activation, promoting IL-1β and IL-18 release.^77^^,^^129^ Mechanistic studies have demonstrated ATP-induced K^+^ efflux and inflammasome assembly.^61^^,^^77^^,^^129^ *In vivo*, silencing or inhibiting P2X7 suppresses NLRP3 activity and alleviates OA inflammation.^130^ In OA models, P2X7 antagonists further reduce synovial hyperplasia and cytokine expression.^129^ These findings directly support its upstream role, while translational inference suggests therapeutic promise.

##### Effects on chondrocytes and cartilage catabolism

In OA models, P2X7 activation experimentally disrupts chondrocyte metabolism and ECM integrity, driving calcium overload, hypertrophy, apoptosis, and catabolic gene expression.^18^^,^^131^ P2X7 activation also induces ER stress in chondrocytes, further impairing cell function (Fig. 3B). Persistent extracellular ATP triggers the unfolded protein response via PERK, IRE1, and ATF6.^18^ Yet, this ER-stress linkage is mainly inferred from broader literature, suggesting that unresolved stress may contribute to OA cartilage degeneration.

##### Role in subchondral bone remodeling and osteoclast activation

P2X7 has been shown in bone biology and OA models to regulate osteoclastogenesis. Normally, data confirm its role in balancing resorption and formation.^96^ Excessive activation is demonstrated *in vitro* and *in vivo* to enhance osteoclast fusion, survival, and bone resorption.^96^^,^^132^ These findings support the inference that dysregulation contributes to subchondral bone loss, sclerosis, and osteophytes in OA.

##### Crosstalk between P2X7 and other signaling pathways in OA

P2X7 receptor activation in OA intersects with multiple inflammatory, metabolic, and stress-related signaling pathways, amplifying joint degeneration and sustaining chronic inflammation. By modulating nuclear factor kappa-B (NF-κB), mitogen-activated protein kinase (MAPK), apoptosis, and P1 receptor signaling, P2X7 establishes a pathological loop that drives synovial inflammation, cartilage catabolism, and bone remodeling.

**Figure 5:**
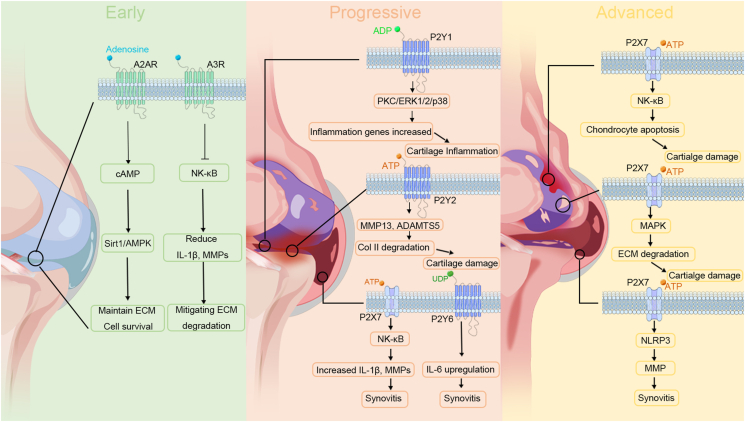
Dynamic purinergic receptor signaling across osteoarthritis stages. The figure illustrates the stage-dependent roles of purinergic receptors in osteoarthritis progression. In the early stage (left), adenosine signaling through A2AR and A3R enhances cAMP–Sirt1/AMPK activity and suppresses NF-κB, thereby maintaining extracellular matrix integrity, promoting chondrocyte survival, and reducing IL-1β and MMPs. During the progressive stage (middle), ADP/ATP-activated P2Y1 and P2Y2 drive PKC/ERK1/2/p38 signaling, the up-regulation of MMP13 and ADAMTS5, and type II collagen degradation, while P2X7 and P2Y6 further stimulate NF-κB and IL-6 secretion, worsening synovitis and cartilage inflammation. In the advanced stage (right), sustained ATP-mediated activation of P2X7 induces NF-κB signaling, chondrocyte apoptosis, MAPK-driven ECM breakdown, and NLRP3 inflammasome activation, leading to extensive MMP release, chronic synovitis, and severe cartilage loss.

In progressive OA, P2X7 acts as a key upstream activator of NF-κB in synoviocytes, macrophages, and chondrocytes, promoting IκBα phosphorylation and degradation, which enables p65/p50 nuclear translocation and the transcription of IL-1β, TNF-α, IL-6, and MMPs (Table 2 and Figure 4, Figure 5).^91^ It also activates MAPK pathways, particularly p38 and c-Jun N-terminal kinase (JNK), enhancing MMP-3, MMP-13, and ADAMTS-5 expression, which accelerates extracellular matrix breakdown and drives irreversible cartilage destruction characteristic of advanced OA (Fig. 5).^133^^,^^134^ The convergence of NF-κB and MAPK signaling promotes synovial hyperplasia and immune infiltration, reinforcing the inflammatory cycle in OA.

Beyond its pro-inflammatory effects, P2X7 signaling intersects with the purinergic P1 receptor axis, particularly the adenosine A3R, which exerts anti-inflammatory and chondroprotective functions by suppressing NF-κB activity and promoting tissue repair.^90^ Overactivation of P2X7 disrupts this balance, aggravating joint degeneration. Similar crosstalk is observed in other contexts, where P2X7 cooperates with A2AR to promote tumor progression, with P2Y2 to amplify inflammasome signaling, and with P2X3/4 to drive inflammatory pain.^135^, ^136^, ^137^, ^138^ Together, these interactions position P2X7 as a central hub within a receptor network where antagonism or synergy shapes inflammation and tissue outcomes, providing a rationale for multi-receptor targeting in OA.

Through its integration with inflammatory transcription factors, stress pathways, and metabolic regulators, P2X7 acts as a central driver of OA progression. It promotes synovial inflammation and cartilage breakdown via NF-κB and MAPK activation, impairs autophagy, increases oxidative stress, and suppresses A3R signaling. This disruption of purinergic homeostasis underscores the need for therapeutic strategies that target both ATP-mediated inflammation and adenosine-mediated repair mechanisms.

#### P2X3 receptor: a critical player in OA pain sensitization

The P2X3 receptor is a homotrimeric ATP-gated ion channel primarily expressed in sensory neurons of the dorsal root ganglia (DRG) and nociceptive fibers that innervate the synovium and subchondral bone. Unlike other P2X receptors found in immune cells and chondrocytes, P2X3 is specialized for pain transmission, acting as a key molecular sensor of ATP released during tissue damage, inflammation, and mechanical stress in OA (Table 2).^126^ In OA, chronic ATP release from inflamed synoviocytes, damaged chondrocytes, and stressed subchondral bone drives peripheral and central sensitization and contributes to chronic OA pain.^123^^,^^126^

##### Role in OA pain transmission

Extracellular ATP levels remain chronically elevated as a consequence of sustained synovial inflammation, progressive cartilage degradation, and persistent mechanical stress, collectively contributing to the prolonged activation of P2X3 and the manifestation of persistent pain, even in the absence of movement. The up-regulation of P2X3 expression in DRG neurons has been associated with increased nociceptor excitability, thereby amplifying afferent pain transmission to the central nervous system.^139^ This sensitization is further exacerbated by pro-inflammatory mediators, which enhance P2X3 responsiveness through the lowering of activation thresholds.^140^^,^^141^ The convergence of these pathological processes positions P2X3 as a central mediator of OA-related hyperalgesia and chronic pain states.

##### P2X3 interaction with pain-related pathways in OA

P2X3 receptors can interact with glutamatergic neurons to promote nerve signal transmission, which is an important mechanism of central sensitization.^126^^,^^142^ ATP-induced P2X3 activation in primary afferents promotes glutamate release at spinal synapses, reinforcing pain transmission in the dorsal horn.^126^ ATP released in the OA joint interacts with bradykinin and prostaglandins, sensitizing P2X3 receptors, amplifying ATP-induced calcium influx, and increasing mechanical pain sensitivity.^123^ The sympathetic nervous system plays a role in chronic OA pain, with ATP released from sympathetic neurons activating P2X3 receptors, contributing to sympathetically maintained pain (SMP), a key feature of chronic OA pain syndromes (Table 4).^126^

**Table 3:** Some agonists and antagonists of purinergic receptors.

Type	Agonist	Impact	Antagonist	Impact
A1R	AST-004^98^	Reduces brain infarction from stroke		
A2AR	CGS21680^79^	Improve cartilage regeneration		
A3R	AST-004^98^	Reduces brain infarction from stroke		
	CF101^90^	Relieve pain		
P2X1	α,β-meATP^115^	Vasoconstriction	NF449^115^	Prevent vasoconstrictions
			Suramin^115^	Prevent vasoconstrictions
P2X2	α,β-meATP^123^	Articular hyperalgesia	A-317491^123^	Relieve articular hyperalgesia
P2X3	α,β-meATP^123^	Articular hyperalgesia	A-317491^123^	Relieve articular hyperalgesia
			Gefapixant^187^	Relieve pain
P2X7			A740003^18^	Reduce cartilage loss
P2Y1	ADP^150^	Activate NLRP3 inflammasome	MRS2179^116^	Inhibit platelets aggregation
	ADPβS^149^	Chondrocyte migration and differentiation		
	MRS2365^66^	Promote platelets activation		
P2Y2			AR-C118925XX^68^	Inhibit cytokine secretion
P2Y4	ATP, UTP^149^	Modulates inflammation and tissue repair		
P2Y6	UDP^20^	Promote the production of inflammatory factors	MRS2578^20^	Inhibit the production of inflammatory factors
P2Y12	ADP^66^	Activates platelets	Clopidogrel^163^	Decrease markers of inflammation
P2Y13	ADPβS^149^	Chondrocyte migration and differentiation		
P2Y14	UDP-G^114^	Increase chemokinesis in eosinophils	PPTN^97^	Reduce osteoblast proliferation

**Table 4:** Some signaling pathways involving purinergic receptors.

Receptor Type	Signaling Pathway	Mechanism	Pathological Outcome	Future Research and Treatment Strategies	Reference
A2AR	Sirt1/AMPK	Chondrocyte homeostasis	Reduce chondrocyte senescence	Develop A2AR agonists to reduce inflammation, preserve cartilage	79
A3R	ROS/NLRP3/GSDMD	Inflammation suppression	Pyroptosis reduction	Target A3AR for anti-inflammatory therapy, relieve pain	90
P2X3	Nociceptive pathway	Nociceptive modulation	Pain perception	Modulate P2X3 to prevent pain hypersensitivity, relieve pain	126
P2X7	RUNX2/ADAMTS5	Upmodulate ADAMTS and MMP	Attenuate cartilage degeneration	Exploring the role of microRNA in P2X7	131
P2Y2	IP3/Ca^2+^	Calcium flux	Osteoblast mechanotransduction	Subchondral bone remodeling	160
P2Y12	PI3K/AKT	Chondrocyte survival	Apoptosis inhibition	Activation of P2Y12 signaling to prevent chondrocyte apoptosis	93

#### Other P2X receptors in OA pathogenesis

Beyond P2X7 and P2X3, other P2X receptors exist, including P2X1, P2X2, P2X4, P2X5, and P2X6. Some of them may also influence OA progression by regulating inflammation, cartilage metabolism, and bone remodeling. These receptors are found in chondrocytes, osteoblasts, osteoclasts, synovial fibroblasts, and immune cells, where they mediate ATP-driven ion fluxes that impact joint function. P2X1 promotes platelet aggregation, an effect associated with microvascular dysfunction and synovial fibrosis.^143^ The presence of P2X4 can promote the activation of inflammasomes mediated by P2X7, thereby promoting the activation of inflammatory factors such as IL-1b (Fig. 4).^144^ In addition, P2X4 in the spinal cord further links it to chronic pain hypersensitivity.^145^

Though less studied than P2X7 and P2X3, other purinergic receptors play emerging roles in mechanotransduction, inflammation, and structural remodeling, highlighting the broader involvement of purinergic signaling in OA. Their activation by mechanical stress suggests that they modulate chondrocyte mechanosensitivity, synovial fibroblast activity, and ECM turnover. Deeper insight into their functions could refine OA signaling models and uncover new therapeutic targets, broadening the scope of purinergic-based interventions.

### P2Y receptors in OA pathogenesis

P2Y receptors are G protein-coupled receptors activated by extracellular nucleotides such as ATP, ADP, UTP, and UDP.^146^ P2Y receptors initiate intracellular signaling pathways that regulate inflammation, cartilage and bone homeostasis, and pain, contributing significantly to OA progression.^147^^,^^148^ Of the eight P2Y subtypes, P2Y1, P2Y2, P2Y4, P2Y6, and P2Y11 couple to Gq or Gs proteins to promote calcium signaling and catabolic responses, while P2Y12, P2Y13, and P2Y14 couple to Gi proteins to suppress cAMP and influence immune and osteoclast activity.^92^^,^^149^, ^150^, ^151^, ^152^

Widely expressed in osteoblasts, osteoclasts, immune cells, and sensory neurons, P2Y receptors regulate cartilage degradation, synovial inflammation, abnormal bone remodeling, and pain sensitization.^147^^,^^153^, ^154^, ^155^ Experimental evidence confirms these functions in cellular and animal OA models, but their role as therapeutic targets remains largely inferential.

#### P2Y1 receptor: modulator of chondrocyte function and synovial activation

P2Y1 activation by ADP leads to intracellular calcium mobilization and downstream activation of the protein kinase C (PKC), ERK1/2, and p38 MAPK pathways, thereby amplifying inflammatory signaling and contributing to cartilage damage during the progressive stage of OA (Figure 4, Figure 5).^156^^,^^157^ In chondrocytes, direct experimental evidence has shown that P2Y1 receptor activation influences cartilage metabolism and matrix turnover. Under physiological conditions, P2Y1 signaling stimulates proteoglycan synthesis and maintains cartilage integrity.^148^ Synovial inflammatory macrophages aggravate neutrophil-mediated joint damage by activating ADP/P2Y1 signaling pathways (Table 2).^153^

#### P2Y2 receptor: mechanosensitive regulator of chondrocyte survival and inflammation

The P2Y2 receptor is a mechanosensitive nucleotide receptor highly expressed in osteocytes.^158^^,^^159^ In OA, animal and *in vitro* studies confirmed that excessive ATP release due to cartilage injury and inflammation leads to chronic P2Y2 activation, triggering catabolic pathways and promoting chondrocyte apoptosis. P2Y2 activation up-regulates MMP-13 and ADAMTS-5 expression, leading to aggrecan and collagen degradation in progressive OA (Figure 4, Figure 5). In osteoblasts and osteocytes, P2Y2 influences mechanotransduction and bone remodeling (Table 2).^155^^,^^158^^,^^160^ Activation of P2Y2 in response to mechanical strain regulates osteocyte-mediated bone resorption and osteoclast differentiation, contributing to subchondral bone loss and structural deterioration in OA.^155^^,^^158^

#### P2Y6 receptors: mediators of synovial inflammation

High levels of UDP have been shown to mediate the proliferation and migration of FLSs through the activation of P2Y6, thereby contributing to synovial hyperplasia and joint inflammation. This conclusion is experimentally validated *in vitro* and in animal models. This activation concurrently induces the up-regulation and secretion of IL-6, a key pro-inflammatory cytokine implicated in the initiation and amplification of arthritic pathogenesis (Fig. 5).^20^ The involvement of the UDP–P2Y6 axis in promoting synovial inflammation highlights its pathological significance in OA. Accordingly, targeted inhibition of P2Y6 signaling has shown efficacy in preclinical models, suggesting its therapeutic potential as a disease-modifying strategy in OA management.

#### Crosstalk and pleiotropic actions of P2Y12 and related receptors

Crosstalk among ADP-sensitive receptors shapes joint homeostasis, with P2Y12 and P2Y13 restraining the osteogenic function of P2Y1, particularly through lipid raft–dependent suppression of calcium signaling.^161^ These mechanisms are supported mainly by cellular experiments, while *in vivo* validation remains limited. In osteoarthritis, P2Y12 plays multifaceted roles: it promotes platelet aggregation and vascular remodeling that may drive synovial fibrosis, yet direct evidence demonstrates that its high expression in chondrocytes protects against IL-1β–induced apoptosis via the PI3K/AKT pathway, thereby delaying cartilage degeneration (Table 4 and Fig. 4).^93^^,^^162^

#### P2Y11 and P2Y14 receptors: regulators of inflammation, bone resorption, and chondrocyte survival

P2Y11 is a dual Gq/Gs-coupled receptor involved in immune modulation and chondrocyte metabolism (Table 5).^163^^,^^164^ Experimental studies have shown that it regulates cAMP production and inflammatory cytokine release, and these effects may have a positive impact on OA treatment (Fig. 3C).^163^^,^^165^ P2Y14 is a UDP-glucose receptor expressed in immune cells, fibroblasts, and chondrocytes, where it regulates glucose metabolism and inflammatory responses (Fig. 4).^71^^,^^166^ Its role in cartilage aging and metabolic dysregulation is largely inferential based on correlative and mechanistic studies rather than direct clinical validation.

**Table 5:** Receptor–drug development stages in purinergic signaling.

Receptor Subtype	Drug	Key Mechanism	Development Stage	Reference
P2X7	Novel allosteric antagonists	Inhibit P2X7 channel activity, intended to dampen inflammatory/neuropathic signaling	*In vitro*	186
	Quercetin (natural product)	Suppresses TRPV1 leading to downregulation of P2X7/NLRP3, shifts macrophage polarization from M1 to M2, reduces Ca^2+^ influx and ATP release	*In vitro*, animal	77
	Probenecid, l-carnitine (repurposed small molecules)	Upregulates miR-373 and inhibits the P2X7/NLRP3/NF-κB axis; lowers IL-1β/IL-18/IL-6/TNF-α	Animal	129
P2X3	Gefapixant (small-molecule antagonist)	Blocks neuronal P2X3 to reduce cough	Clinical	189
P2Y6	UDP (endogenous agonist)	Promotes RA fibroblast-like synoviocyte proliferation/migration and IL-6 secretion	*In vitro*, animal	20
	MRS2578 (small-molecule antagonist)	Antagonizes UDP to suppress FLS-driven inflammation and CIA progression	*In vitro*, animal	20
P2Y12	Selatogrel	Rapid, potent, and reversible inhibition of platelet P2Y12	Clinical	193
P2Y11	NF340	Activation induces IL-6/IL-8 and sTNFR2 release, suppresses TLR4-driven TNF-α secretion	*In vitro*	164
P2	Suramin (non-selective)	Activates Nrf2 and inhibits NF-κB/MAPK, anti-inflammatory, anti-MMP/ADAMTS, promotes matrix synthesis	Clinical	191
A2AR	CGS21680	Maintains cartilage homeostasis: anti-senescence, pro-autophagy, mitochondrial protection	*In vitro*, animal	79
A3R	CF101	Suppresses ROS/NLRP3/GSDMD pyroptosis pathway, reduces cartilage degradation and pain	*In vitro*, animal	90

### P1 receptor signaling and its chondroprotective effects

P1 receptor signaling counteracts inflammation, oxidative stress, and chondrocyte apoptosis in OA via adenosine-mediated activation of A2A, A3, and A2B receptors.^74^^,^^79^^,^^90^ In OA, disrupted adenosine metabolism and impaired P1 signaling exacerbate synovial inflammation, ECM degradation, and cartilage loss. A2AR serves as a key regulator of chondrocyte survival and metabolism, while A3 inhibits inflammation-induced cartilage damage. A2BR has dual roles, contributing to cartilage repair but also promoting synovial fibrosis and inflammation. Elucidating these distinct P1 receptor functions offers valuable therapeutic insights for OA management.

#### A2A receptor: a master regulator of chondrocyte survival

The A2A adenosine receptor (A2AR) is a Gs-coupled purinergic receptor that plays a critical role in chondrocyte survival and mitochondrial homeostasis in osteoarthritis (OA).^15^^,^^79^ It is widely expressed in chondrocytes and osteoblasts, where it regulates autophagy, oxidative stress responses, and extracellular matrix (ECM) metabolism.^16^^,^^79^ In OA, A2AR expression is down-regulated, impairing its protective and anti-inflammatory functions, leading to chondrocyte apoptosis, mitochondrial dysfunction, and progressive cartilage degradation.

A2AR activation enhances cAMP production, triggering downstream protective signaling pathways, including Sirt1/AMPK, FoxO, and PI3K/Akt, which collectively modulate autophagy, suppress inflammation, and preserve mitochondrial homeostasis, thereby maintaining cartilage integrity in the early stage of OA (Table 4 and Figure 4, Figure 5).^16^^,^^79^ It also inhibits NF-κB and MAPK signalling, suppressing pro-inflammatory cytokines and catabolic enzymes (Fig. 4).^167^^,^^168^ These findings are supported by *in vivo* mouse models and *in vitro* chondrocyte experiments. However, their translation to clinical OA remains inferential. Given its broad regulatory role, A2AR is regarded as a key therapeutic target for OA, with its agonists showing potential disease-modifying effects.

##### A2AR activation and autophagy regulation

Autophagy is a crucial cellular quality control mechanism that maintains chondrocyte homeostasis by degrading damaged organelles, protein aggregates, and reactive oxygen species (ROS).^16^^,^^169^ In OA, defective autophagy leads to chondrocyte senescence and apoptosis, contributing to cartilage matrix degradation and joint degeneration. The experimental data confirmed that A2AR activation enhances autophagy flux via the Sirt1/AMPK and FoxO pathways in chondrocytes (Fig. 3A).^16^^,^^79^ Clinical evidence, however, is still lacking.

A2AR is a key upstream modulator of AMP-activated protein kinase (AMPK), a central regulator of cellular energy homeostasis. Under stress, AMPK promotes ATP generation and inhibits anabolic pathways. In OA, this adaptive axis is impaired, marked by reduced AMPK activity, leading to energy imbalance, heightened catabolism, and cartilage degeneration.^170^^,^^171^ A2AR also activates the FoxO1 and FoxO3 transcription factors, which regulate chondrocyte viability by controlling oxidative stress, autophagy, and ECM synthesis.^172^ These mechanisms are demonstrated in preclinical studies, but their clinical validation is still inferential. A2AR stimulation enhances FoxO nuclear translocation and upregulates genes such as SOD2 and catalase.^16^ In OA, diminished FoxO activity disrupts redox balance and accelerates matrix loss, but A2AR activation restores FoxO function, mitigates oxidative damage, and preserves cartilage, highlighting the therapeutic potential of targeting the A2AR–AMPK–FoxO axis.

##### Anti-inflammatory and crosstalk roles of A2AR in OA

A2AR activation suppresses TNF-α-induced MMP-3 expression, thereby preventing ECM degradation and collagen breakdown. This effect has been experimentally validated in cell and animal models. In OA, elevated levels of MMP-3, MMP-13, and ADAMTS-5 contribute to aggrecan and type II collagen cleavage, compromising cartilage integrity.^173^ A2AR stimulation down-regulates catabolic enzymes and up-regulates anabolic factors, preserving cartilage structure.^78^ In the synovium and fibroblast-like synoviocytes (FLSs), A2AR expression inversely correlates with arthritis severity and FLS activation.^174^ It also promotes M2 macrophage polarization, shifting immune responses away from the pro-inflammatory M1 phenotypes and thereby reducing joint inflammation.^175^

A2AR-deficient mice exhibit severe cartilage loss, mitochondrial dysfunction, ROS accumulation, and heightened inflammation, underscoring its role in joint homeostasis (Table 2).^15^ A2AR agonists reduce inflammation, restore mitochondrial function, and protect cartilage in OA models.^15^^,^^16^^,^^79^ These data are experimentally robust, but no clinical trials have confirmed their therapeutic efficacy. Beyond these anti-inflammatory functions, A2AR also engages in crosstalk with other purinergic receptors. The reported interactions with A1R and P2X7R are mainly derived from neuroscience and immunology studies, and their relevance to OA remains largely inferential.^176^^,^^177^ This interplay illustrates how A2AR not only exerts direct anti-catabolic and immunomodulatory effects but also acts as a central hub balancing inhibitory and pro-inflammatory signals within the purinergic network.

#### A3 receptor: a potent inhibitor of OA inflammation

The A3 adenosine receptor (A3R) is a Gi-coupled purinergic receptor that is primarily involved in inflammatory resolution by inhibiting inflammatory factor expression.^178^ In OA, A3R expression is often reduced, impairing its protective effects against inflammatory cytokine release and chondrocyte apoptosis.

In early OA, A3R activation exerts potent anti-inflammatory effects by suppressing NF-κB and NLRP3 inflammasome activity, thereby reducing pro-inflammatory cytokines, MMPs, and oxidative stress mediators (Fig. 5). A key mechanism involves the inhibition of the NLRP3 inflammasome (Fig. 4), a major driver of sterile inflammation in OA that regulates IL-1β and TNF-α production, contributing to synovitis, cartilage degradation, and osteoclastogenesis.^179^^,^^180^ A3R also blocks NF-κB nuclear translocation, limiting IL-1β and TNF-α transcription and attenuating immune cell infiltration, synovial hyperplasia, and ECM breakdown (Fig. 5). Additionally, A3R activation enhances MAPK signaling, which may influence fibroblast proliferation.^181^

#### A2B receptor in OA with dual roles

A2BR plays a dual role in OA by supporting cartilage repair and homeostasis while also promoting synovial fibrosis and sustaining chronic inflammation, thereby contributing to disease progression. The beneficial effects on bone marrow mesenchymal stem cell (BMSC) osteogenic differentiation have been experimentally validated (Fig. 4).^182^^,^^183^ However, the contribution of A2BR to synovial fibrosis and angiogenesis is primarily inferential based on cytokine secretion patterns.^184^ This underscores the need for context-dependent modulation to capture reparative benefits while minimizing fibrotic and pro-angiogenic liabilities.

Collectively, the opposing roles of P2 and P1 receptors illustrate the dynamic balance between pro-inflammatory, catabolic signaling and anti-inflammatory, chondroprotective mechanisms in OA. Understanding this molecular interplay provides a foundation for therapeutic interventions aimed at targeting purinergic receptors to modulate disease progression, alleviate pain, and restore joint homeostasis. The next section explores the therapeutic implications of targeting purinergic receptors in OA, focusing on pharmacological interventions, gene modulation strategies, and translational approaches for clinical application.

### Purinergic receptor modulation in OA with challenges and prospects

Modulation of purinergic receptor activity, through either activation or inhibition, has been investigated as a potential disease-modifying strategy in OA, with the capacity to influence inflammation, cartilage metabolism, and pain. Yet, translation into clinical use remains constrained by pharmacological limitations and an incomplete understanding of receptor dynamics within complex joint environments. Advances in innovative research approaches, capable of capturing the cellular diversity and tissue context, are expanding insights into how purinergic signaling shapes OA pathogenesis. These developments provide a foundation for precision medicine strategies that tailor receptor targeting to patient-specific features and disease stages, opening new avenues for therapeutic intervention.

### Targeting P2X receptors: suppressing inflammation and pain sensitization

The P2X receptor family plays a central role in OA pathogenesis by mediating ATP-driven inflammation, cartilage degradation, and pain sensitization. Among these, P2X7 and P2X3 have emerged as key therapeutic targets due to their involvement in synovial inflammation and nociceptive transmission. Excessive activation of these receptors perpetuates chronic joint inflammation and pain, making them attractive candidates for pharmacological intervention.

#### P2X7 antagonists: reducing synovial inflammation and cartilage breakdown

P2X7 is a well-characterized purinergic receptor in OA, implicated in synovial inflammation, oxidative stress, and chondrocyte apoptosis. Its activation triggers NLRP3 inflammasome assembly and subsequent IL-1β and TNF-α release, amplifying inflammatory and catabolic pathways (Table 5).^77^^,^^129^ Sustained P2X7 signaling also induces calcium influx and ER stress, exacerbating chondrocyte death.^18^ In subchondral bone, it promotes osteoclastogenesis and pathological remodeling, contributing to joint stress and cartilage degradation.^132^ P2X7 antagonists have faced major translational hurdles, as most candidates suffer from inadequate pharmacokinetics, limited brain penetration, and poor selectivity, which collectively undermines their clinical efficacy despite strong preclinical promise (Table 5).^185^

#### P2X3 antagonists: modulating OA pain

P2X3 plays a key role in OA pain sensitization by mediating ATP-driven nociceptive signaling. Its persistent activation increases neuronal excitability, hyperalgesia, and central sensitization, intensifying pain perception. Gefapixant, a selective P2X3 antagonist, has shown efficacy in treating neuropathic pain and chronic cough, with potential applications in OA pain currently under investigation (Table 3).^186^ Gefapixant's development is constrained by a narrow therapeutic window, where clinically relevant efficacy at higher doses is offset by frequent taste-related adverse events that limit patient adherence (Table 5).^187^^,^^188^ Targeting P2X3 alleviates nociceptive hypersensitivity, while concurrent activation of A2A or A3 receptors enhances chondroprotection and suppresses inflammation, thereby combining symptomatic relief with structural preservation.

### Targeting P2Y receptors: modulating joint inflammation and bone remodeling

P2Y receptors regulate intracellular signaling cascades that influence inflammation, cartilage metabolism, and bone remodeling. The selective targeting of these receptors may provide novel strategies for modulating OA progression while preserving essential physiological functions.

#### P2Y2 and P2Y6 inhibitors: preventing synovial hyperplasia and cartilage breakdown

Blocking P2Y2 and P2Y6 receptors has emerged as a potential strategy for limiting cartilage degeneration and synovial inflammation in OA. Experimental studies have shown that pharmacological inhibition of P2Y2 prevents chondrocyte hypertrophy and reduces inflammatory cytokine production, preserving cartilage integrity. The broad-spectrum P2 receptor antagonist suramin can promote the polarization of M1 macrophages to M2 macrophages and reduce the inflammatory response and extracellular matrix degradation.^189^ Similarly, MRS2578, a P2Y6 antagonist, has been shown to reduce IL-6 secretion and alleviate synovial inflammation (Table 3, Table 5).^20^ The clinical advancement of suramin and MRS2578 is constrained by suramin's excessively prolonged half-life with poor clearance and MRS2578's paradoxical exacerbation of ischemic injury through impaired microglial phagocytosis (Table 5).^190^^,^^191^

#### P2Y12 and P2Y11 modulators: protecting cartilage and reducing fibrosis

P2Y12 receptor activation has been shown to protect against chondrocytes apoptosis by activating the PI3K/AKT pathway, which enhances cell survival.^93^ Pharmacological activation of P2Y12 may offer a potential strategy for preserving cartilage viability and delaying OA-related structural deterioration. Drug blocking or knocking out P2Y11 can regulate the release of inflammatory factors and alleviate the inflammatory response by regulating cAMP in macrophages, which may become a potential strategy for the treatment of OA.^163^ Receptor modulators face translational and clinical constraints primarily due to their pharmacokinetic dependence on absorption and metabolic activation, suboptimal pharmacodynamic efficacy, and a substantial bleeding liability in vulnerable populations (Table 5).^192^

### Targeting P1 receptors: enhancing cartilage protection and repair

P1 receptor activation exerts anti-inflammatory, anti-apoptotic, and anabolic effects in OA, making it an attractive target for disease-modifying interventions. Among the four P1 receptor subtypes, A2A and A3 receptors have demonstrated the most promising chondroprotective and anti-inflammatory properties, while A2BR signaling plays a dual role in cartilage repair and fibrosis. Modulating these receptors with selective agonists offers a potential therapeutic approach to suppress joint inflammation, enhance chondrocyte survival, and promote cartilage regeneration.

#### A2A and A3 receptor agonists: protecting cartilage and suppressing inflammation

A2AR regulates chondrocyte survival, autophagy, and oxidative stress resistance by activating the Sirt1/AMPK and FoxO pathways, which enhance mitochondrial function and promote chondrocyte longevity (Table 3, Table 5).^16^^,^^79^ A2AR activation also mitigates oxidative damage and mitochondrial dysfunction, which are key contributors to chondrocyte apoptosis.^15^ A3R exerts anti-inflammatory effects by inhibiting the NLRP3 inflammasome, thereby reducing IL-1β and TNF-α release in OA joints (Table 2, Table 4).^90^ A3R activation also alleviates joint pain and cytokine production, fostering a reparative microenvironment.^178^ CGS21680, a selective A2A agonist, protects cartilage, reduces swelling, and promotes cell proliferation.^78^ CF101 (IB-MECA), a selective A3 agonist, attenuates cartilage damage and pain, offering potent chondroprotection (Table 3, Table 5).^90^ The principal bottleneck for this receptor modulator stems from its suboptimal clinical efficacy and the necessity for rigorous monitoring of systemic adverse effects, both of which restrict its therapeutic utility. Importantly, combination strategies may broaden the therapeutic scope, as P2X7 inhibition curtails inflammasome-driven inflammation, while A2A or A3 activation strengthens chondroprotective and anti-inflammatory pathways, together supporting durable disease modification.

### Technological and therapeutic frontiers in purinergic receptor research

Despite the therapeutic promise of targeting purinergic receptors in OA, several translational hurdles remain. The dual roles of certain receptors in both protective and pathogenic processes demand precise, context-dependent modulation. Advances in drug design, gene modulation, and targeted delivery offer opportunities to refine these therapies, while emerging platforms such as single-cell transcriptomics, spatial transcriptomics, and spatial proteomics provide unprecedented resolution to define receptor heterogeneity, microenvironmental context, and patient-specific signaling profiles. Nonetheless, challenges related to specificity, systemic side effects, and interpatient variability must be addressed to ensure clinical success.

#### Receptor biology in advanced single-cell and spatial technologies

Advanced receptor studies show how new technologies reshape mechanistic insight and therapeutic strategies. Single-cell transcriptomics revealed P2Y6-driven immune regulation in lung adenocarcinoma (LUAD), while computational analysis with immunofluorescence clarified P2Y12 shifts in ischemic microglia.^193^^,^^194^ In pancreatic ductal adenocarcinoma (PDAC), imaging mass cytometry and mass spectrometry imaging exposed the spatial heterogeneity of extracellular adenosine and receptor-rich niches linked to therapy resistance.^195^ Together, these findings emphasize that receptor biology, rather than bulk expression, must be examined within its microenvironment. Single-cell and spatial methods provide decisive advantages: transcriptomics uncovers cell-type–specific receptor variation, spatial transcriptomics retains the anatomical context, and spatial proteomics supplies protein-level validation. Their integration resolves receptor heterogeneity, maps intercellular signaling, and pinpoints spatially confined vulnerabilities, enabling precision immunotherapy and more context-aware receptor-targeted therapies.

#### Selective targeting strategies for purinergic receptors

A key challenge in purinergic-based OA therapy is achieving high receptor specificity, given the widespread expression of purinergic receptors across various tissues and organ systems. Future therapeutic strategies should prioritize receptor-selective approaches to minimize off-target effects and enhance clinical safety. These include the development of allosteric modulators, which bind to non-orthosteric sites on the receptor and allow for fine-tuned modulation of receptor activity without interfering with endogenous ligand binding or disrupting normal physiological function. Additionally, biased agonists offer a promising avenue by selectively activating beneficial downstream signaling pathways while avoiding those linked to adverse effects, thereby enhancing therapeutic precision and efficacy.^196^

#### Advanced drug delivery systems for purinergic receptor modulation

Effective clinical translation of purinergic receptor-based therapies requires optimized drug delivery for localized and sustained receptor modulation. Systemic drug administration is limited by poor bioavailability, rapid metabolism, and off-target effects. Nanoparticles enhance drug stability and retention in joint tissues, enabling the precise delivery of purinergic modulators. Lipid- and polymer-based nanoparticles have been used to deliver P2X7 antagonists and A2AR agonists directly to inflamed joints, improving efficacy and minimizing systemic exposure.^79^^,^^197^

#### Personalized medicine: optimizing therapy based on patient-specific purinergic signaling profiles

Given the heterogeneity of OA in inflammation, cartilage loss, and pain sensitization, personalized purinergic receptor modulation may enhance treatment efficacy. Biomarkers reflecting purinergic signaling activity could support stratified therapeutic approaches tailored to individual disease phenotypes. Advances in single-cell transcriptomics and proteomics have revealed purinergic receptor expression patterns across OA tissues.^198^^,^^199^ Diagnostic tools assessing receptor levels in synovial fluid or cartilage biopsies may enable precision medicine strategies.

## Conclusion

OA is a progressive joint disease driven by mechanical, inflammatory, and metabolic factors, yet current treatments remain largely symptomatic. Purinergic receptors, including adenosine-responsive P1 and extracellular nucleotide-activated P2 families, are central regulators of joint homeostasis. In OA, excessive P2 activation promotes inflammation, cartilage breakdown, and pain, while P1 signaling confers chondroprotection and immune resolution. Modulating these pathways through receptor activation or inhibition offers a promising disease-modifying approach. Although challenges remain due to receptor complexity and systemic distribution, advances in selective targeting, innovative delivery systems, and next-generation profiling technologies are paving the way for precision strategies. By integrating these innovations, purinergic receptor modulation may evolve into a clinically viable therapy to slow progression, preserve cartilage, and improve patient outcomes.

## CRediT authorship contribution statement

**Hongliang Li:** Writing – original draft, Resources, Formal analysis, Data curation. **Tianqi Wang:** Writing – review & editing, Supervision, Project administration, Investigation, Formal analysis, Data curation, Conceptualization. **Zi Wang:** Validation, Data curation. **Jincen Hou:** Visualization, Validation. **Zhong Li:** Supervision, Methodology, Investigation. **Jiyuan Yan:** Writing – review & editing, Supervision, Resources, Project administration, Funding acquisition, Conceptualization.

## Data availability statement

The data that support the findings of this study are available from the corresponding authors upon reasonable request.

## Funding

This work was financially supported by Sichuan Science and Technology Program (China) (No. 2022YFS0628) to Jiyuan Yan and the Doctoral Research Initiation Fund of Affiliated Hospital of Southwest Medical University to Jiyuan Yan.

## Conflict of interests

The authors declare that they have no conflict of interests.
